# Body composition has limited influence on the eGFRcys/eGFRcrea ratio in older women

**DOI:** 10.1093/ckj/sfag379

**Published:** 2026-10-28

**Authors:** Rona Dawar, Anders Christensson, Kristina E Åkesson, Agne Laucyte-Cibulskiene, Linnea Malmgren

**Affiliations:** Department of Clinical Sciences, Lund University, Malmö, Sweden; Department of Nephrology, Skåne University Hospital, Malmö, Sweden; Department of Clinical Sciences, Lund University, Malmö, Sweden; Department of Nephrology, Skåne University Hospital, Malmö, Sweden; Department of Clinical Sciences, Orthopedics, Lund University, Malmö, Sweden; Department of Orthopedics, Skåne University Hospital, Sweden; Department of Clinical Sciences, Lund University, Malmö, Sweden; Department of Nephrology, Skåne University Hospital, Malmö, Sweden; Department of Clinical Sciences, Lund University, Malmö, Sweden; Department of Geriatrics, Skåne University Hospital, Malmö, Sweden

**Keywords:** chronic kidney disease, creatinine, cystatin C, estimated glomerular filtration rate, SGHS

## Abstract

**Background:**

Kidney function is best estimated using both creatinine- and cystatin C-based estimated glomerular filtration rate (eGFR). A low ratio between eGFR based on cystatin C and eGFR based on creatinine (eGFR_cys_/eGFR_crea _ratio <0.7), termed selective glomerular hypofiltration syndromes, is associated with increased cardiovascular risk. While differences in filtration for molecules with different molecular masses may explain this, non-renal factors such as body composition have also been proposed. We aimed to assess whether body composition determines the eGFR_cys_/eGFR_crea_ ratio in older women.

**Methods:**

In 1044 women aged 75 from the Osteoporosis Prospective Risk Assessment (OPRA) cohort, followed to ages 80 (*n* = 715) and 85 (*n* = 382), we analyzed associations between the eGFR_cys_/eGFR_crea_ ratio and body composition (fat and appendicular lean mass), muscle strength (knee extension, Nm), and sarcopenia [European Working Group on Sarcopenia in Older People 2 (EWGSOP2) criteria]. Adjusted *R*² was reported from linear regression model assessing the eGFR_cys_/eGFR_crea_ ratio as the dependent variable adjusted for high-sensitivity C-reactive protein (hsCRP), smoking, and steroid treatment.

**Results:**

Associations between body composition measures and the eGFR_cys_/eGFR_crea_ ratio were generally low and attenuated with age. No significant association was observed between fat mass and the ratio at any age. Muscle mass was positively associated with the ratio at ages 75 and 80, but not at age 85. Muscle strength was consistently associated with the eGFR_cys_/eGFR_crea_ ratio across all ages, although the proportion of explained variance declined with increasing age (adjusted *R*^2^ 7.4%, 6.0%, and 2.4% at ages 75, 80, and 85, respectively). Sarcopenia was inversely associated with the ratio at ages 75 and 85, but not at age 80.

**Conclusion:**

Body composition, muscle strength, and sarcopenia showed only weak associations with the eGFR_cys/_eGFR_crea_ ratio in older women. These findings suggest that non-body composition factors, including intrinsic renal mechanisms such as selective filtration of larger molecules, may contribute to a low eGFR_cys_/eGFR_crea_ ratio.

## Graphical Abstract

**Graphical Abstract:**
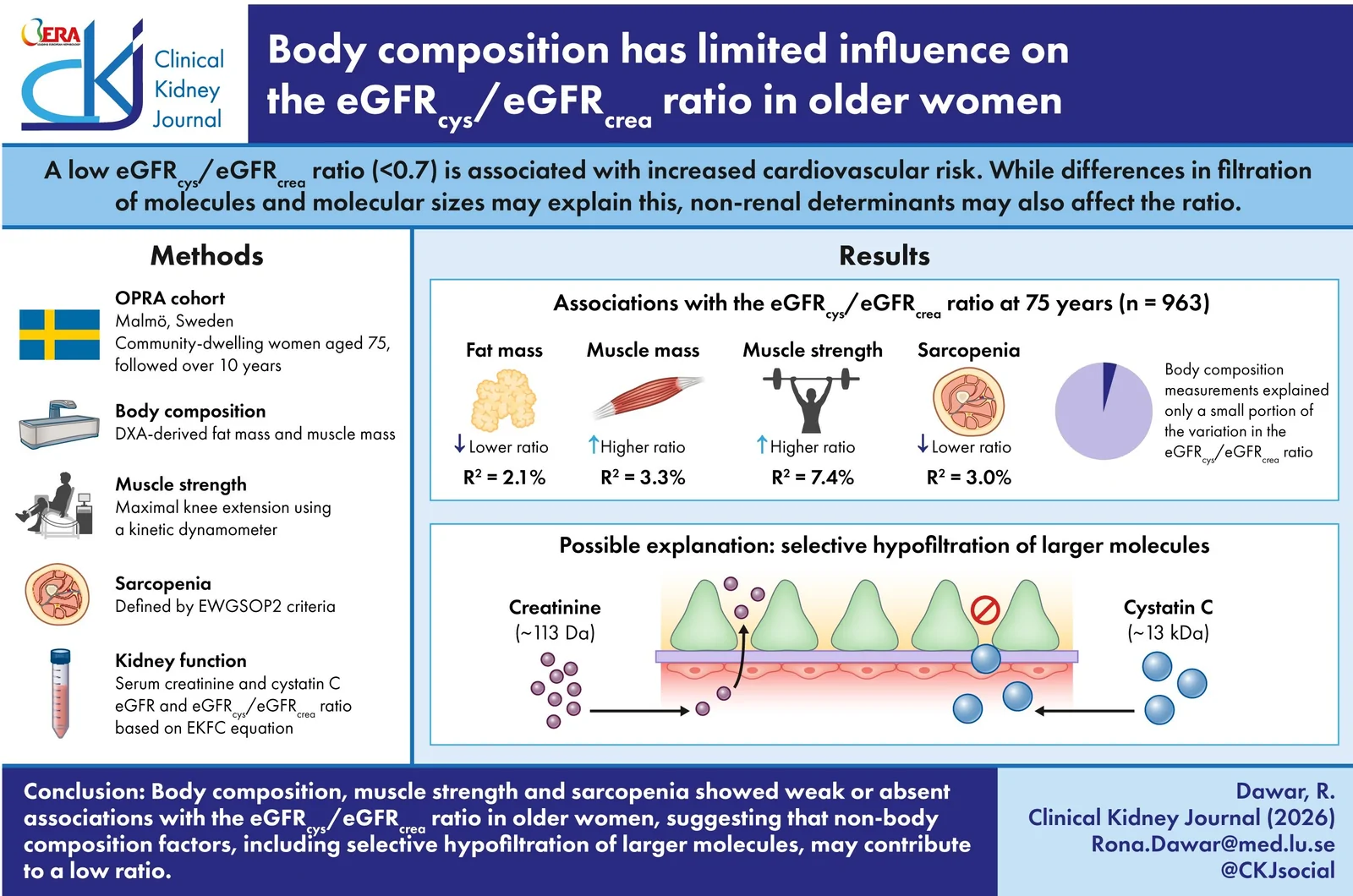

KEY LEARNING POINTS **What was known:**

- A low ratio between cystatin C-based and creatinine-based estimated glomerular filtration rate (eGFR_cys_/eGFR_crea _ratio) has been proposed as a marker of selective glomerular hypofiltration syndromes, but the influence of non-renal determinants on the ratio remains incompletely understood.
- Creatinine and cystatin C are affected by different non- glomerular filtration rate (GFR) determinants, including muscle mass, adiposity, inflammation, and glucocorticoid treatment, which may contribute to discrepancies between filtration estimates.
- Data on the association between body composition, muscle strength, sarcopenia, and the eGFR_cys_/eGFR_crea_ ratio are limited.

**This study adds:**

- Body composition, muscle strength, and sarcopenia were only weakly associated with the eGFR_cys_/eGFR_crea_ ratio in community-dwelling older women from the Osteoporosis Prospective Risk Assessment (OPRA) cohort.
- Although some associations were observed, these factors explained only a small proportion, <10%, of the variability in the ratio across all examined age groups.
- Our findings suggest that non-renal determinants related to body composition alone are unlikely to fully explain a low eGFR_cys_/eGFR_crea_ ratio in older women.

**Potential impact:**

- These findings support the continued clinical relevance of a low eGFR_cys_/eGFR_crea_ ratio as a potential marker of altered glomerular filtration beyond differences in body composition.
- Clinicians should be aware that muscle mass and muscle strength, but not fat mass, showed weak but consistent associations with the eGFR_cys_/eGFR_crea_ ratio across the primary and sensitivity analyses, although their overall contribution appears limited in older women.
- Future studies should further investigate intrinsic renal mechanisms and other non-GFR determinants underlying discrepancies between cystatin C- and creatinine-based filtration estimates.

## INTRODUCTION

Chronic kidney disease (CKD) affects a substantial portion of the global population, placing individuals at increased risk of cardiovascular disease (CVD) and mortality [1, 2]. CKD is commonly assessed using estimated glomerular filtration rate (eGFR), derived from creatinine (eGFR_crea_) and/or cystatin C (eGFR_cys_), together with albuminuria. Compared with creatinine, cystatin C provides a more reliable estimate of kidney function, while combined creatinine- and cystatin C-based eGFR provide the most accurate assessment [3–5]. In addition, eGFR_cys_ more strongly predicts CVD and mortality than eGFR_crea_ [6–8]. Although, the mechanisms underlying this difference remain incompletely understood. The eGFR_cys_/eGFR_crea_ ratio has emerged as a means of capturing discrepancies between these estimates. An eGFR_cys_/eGFR_crea_ ratio <0.7, termed selective glomerular hypofiltration syndrome (SGHS; formerly shrunken pore syndrome), occurs in ~10% of the population, and is a strong predictor of cardiovascular risk and mortality independent of kidney function [9–14].

SGHS has been proposed to reflect selective impairment in the filtration of larger molecules, such as cystatin C (≈13 kDa), relative to smaller molecules like creatinine (113 Da), resulting in a disproportionate increase in plasma cystatin C and reduced eGFR_cys_/eGFR_crea_ ratio independent of overall kidney function [11, 13]. This association may also reflect alterations in the circulating proteome, including accumulation of pro-atherogenic signalling proteins [15–18].

Discrepancies between eGFR_cys_ and eGFR_crea_ may alternatively reflect non-renal influences. Cystatin C concentrations may increase with inflammation, systemic steroid treatment, hyperthyroidism, and higher fat mass (FM), whereas creatinine is influenced by muscle mass, physical activity, and dietary protein intake [17, 19–22]. Consequently, differences in body composition may distort the eGFR_cys_/eGFR_crea_ ratio independent of kidney function (Fig. 1). This interpretation is proposed by previous studies suggesting that discrepancies between the eGFR_cys_ and eGFR_crea_ and may partly reflect confounding by non-GFR determinants rather than selective glomerular filtration [23–25].

**Figure 1:**
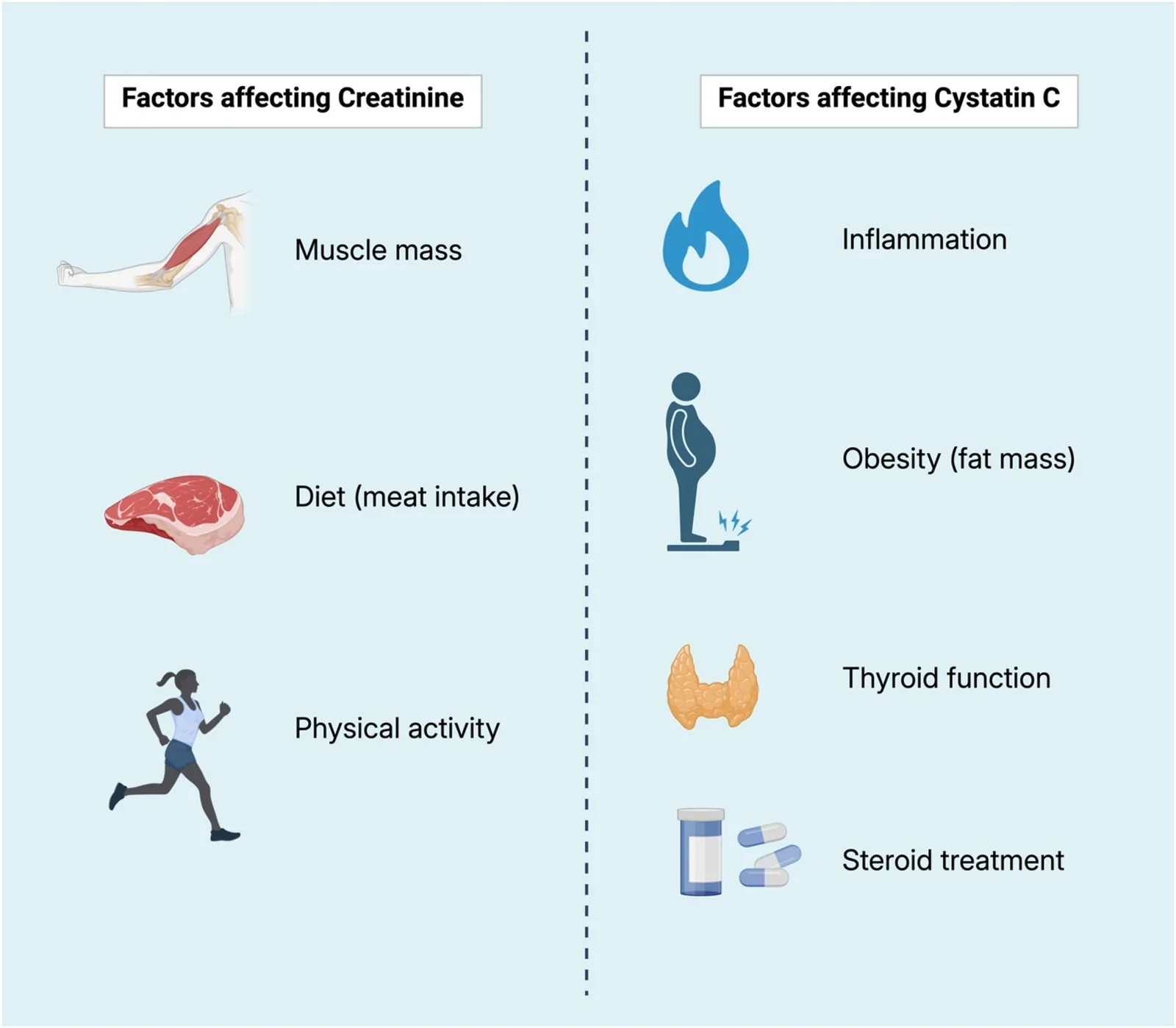
Factors affecting creatinine and cystatin C levels. Created with BioRender.com.

Most studies examining the relationship between creatinine and muscle mass have been conducted in young populations, whereas this relationship remains poorly investigated in older women [21, 26, 27], who experience pronounced age-related changes in muscle mass and body composition associated with frailty and morbidity [28, 29]. Such changes may alter circulating levels of cystatin C and creatinine, potentially contributing to misclassification of SGHS in older women [30–32]. We therefore aimed to evaluate whether body composition influences the eGFR_cys_/eGFR_crea_ ratio in older women by examining the association with FM, muscle mass, muscle strength, and sarcopenia in a population-based cohort of 1044 women aged 75 years at baseline, followed for 10 years. Based on the growing body of evidence supporting the concept of selective glomerular hypofiltration, whereby reduced clearance of larger molecules such as cystatin C results in a lower eGFR_cys_/eGFR_crea_ ratio, we hypothesized that body composition measures alone would not explain the variation in the ratio.

## MATERIALS AND METHODS

### Subjects

In Malmö, Sweden, between 1995 and 1999, 1604 randomly selected 75-year-old women were invited to participate in the Osteoporosis Prospective Risk Assessment (OPRA) study. No exclusion criteria were applied [33]. 1044 women (65% response rate) were included at baseline. Follow-up investigations were conducted after 5 (*n* = 715) and 10 years (*n* = 382), at ages 80 and 85, respectively (Fig. 2). Women with available cystatin C and creatinine measurements were included, totaling 963 participants at baseline, 683 at the 5-year follow-up, and 355 the 10-year follow-up [12]. Mortality data were obtained from the Swedish National Population Register.

**Figure 2:**
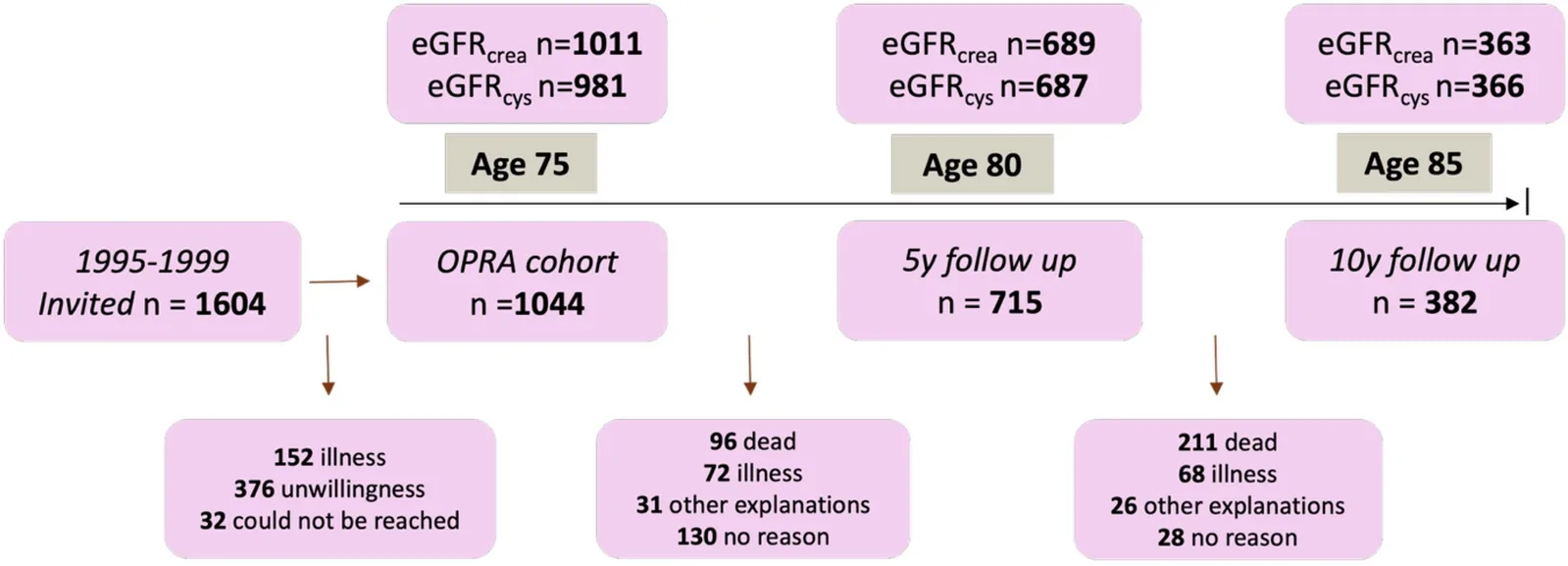
Flowchart of study participants in the OPRA cohort across baseline and follow-up visits.

The study was approved by the Regional Ethical Review Board in Lund (Dnr: LU200-95 and LU2014804) and performed in accordance with the revised Helsinki declaration. All participants provided written informed consent.

### General biochemistry

Analyses were performed at the Department of Clinical Chemistry, Skåne University Hospital, Sweden. Blood samples were collected non-fasting before noon and stored at −80°C. Detailed methods for cystatin C and creatinine measurements have been reported previously [33]. Plasma cystatin C was analyzed in batches in 2015 using a Cobas analyzer calibrated to the ERM-DA471/IFCC reference standard (Roche Diagnostics). Plasma creatinine was measured using the Beckman Synchron LX20-4 or Cobas system and standardized to the IDMS reference method [35]. Plasma high-sensitivity C-reactive protein (hsCRP) was measured according to routine clinical laboratory procedures [34].

### Kidney function and the eGFR_cys_/eGFR_crea_ ratio

eGFR was calculated using the European Kidney Function Consortium (EKFC) equations based on creatinine (eGFR_crea_) and cystatin C (eGFR_cys_) for females, given their established performance in European older-adult populations [35, 36]. The eGFR_cys_/eGFR_crea_ ratio was calculated by dividing eGFR_cys_ by eGFR_crea_. Currently, values <0.6 or <0.7 have been proposed to define SGHS [13, 18]. Sensitivity analyses used two additional equation pairs: the revised Lund–Malmö (LM-rev) and Caucasian, Asian, Pediatric and Adult (CAPA) equations, and the creatinine- and cystatin C-based CKD Epidemiology Collaboration (CKD-EPI, 2009 and 2012) equations. The corresponding eGFRcys/eGFRcrea ratios were recalculated for each pair (Tables S2a–c and S3a–c).

### Body composition, muscle strength, and sarcopenia

Participants underwent dual-energy X-ray absorptiometry (DXA), anthropometrics, and isokinetic muscle strength testing at each follow-up. Total lean mass (TLM), appendicular lean mass (ALM), and FM were measured using DXA. TLM, expressed in grams, includes all lean soft tissue and may overestimate skeletal mass; ALM was therefore used instead, as appendicular regions predominantly contain skeletal muscle. To account for body size, ALM was expressed as the ALM index (ALM divided by height squared, kg/m^2^) and used as a proxy for skeletal muscle mass [37, 38]. FM (kg) was used as a measure of total body adiposity.

Muscle strength was assessed as maximal knee extension [Newton meters (Nm)] using an isokinetic dynamometer [38]. Sarcopenia was defined according to European Working Group on Sarcopenia in Older People 2 (EWGSOP2) criteria as low muscle strength plus low muscle mass [39]. Low muscle strength was defined as knee extension strength <175 Nm, whereas low muscle mass was defined as an ALM/height^2^ <5.5 kg/m^2^.

### Statistics

Demographic data are presented as mean with standard deviations (SDs) or median (interquartile range). Separate cross-sectional linear regression analyses were performed, with the EKFC-based eGFR_cys_/eGFR_crea_ ratio used as the dependent variable. FM, muscle mass, and muscle strength were analyzed as continuous variables; sarcopenia was categorical (yes/no). The regression model (Model 1) was adjusted for smoking, hsCRP, and steroid treatment (Fig. 1). A second model (Model 2), additionally adjusted for thyroid-stimulating hormone (TSH), was applied to the age-75 analyses only, as TSH was only available at baseline. Analyses were repeated at ages 80 (*n* = 683) and 85 (*n* = 355). Sensitivity analyses used the LM-rev, CAPA, and CKD-EPI equations, with ratios recalculated accordingly. Separate regression analyses used eGFRcrea and eGFRcys individually as dependent variables. Results are presented as regression coefficients (*β*) with 95% CI, adjusted *R*^2^, and a significance threshold of *P* < .05. Differences in the eGFR_cys_/eGFR_crea_ ratio between sarcopenic and non-sarcopenic individuals were assessed using independent sample *t*-tests (two-tailed) at each age.

All statistical analyses were made using SPSS (IBM Corp. Released 2020. IBM SPSS Statistics for Windows, Version 27.0. Armonk, NY).

## RESULTS

At baseline, the 963 women had a mean age of 75.2 (0.1) years. Antihypertensive medication was used by 321 (33%) women, thyroid hormone by 121 (12.5%), 151 (18%) had CVD, and 53 (6%) had diabetes mellitus. The mean eGFRcys/eGFRcrea ratio was 0.98 (Table 1). An eGFR_cys_/eGFR_crea_ ratio <0.6 was present in 23 (2.3%) women and a ratio <0.7 in 64 (6.6%) women. Similar proportions were observed at ages 80 and 85.

**Table 1:** Characteristics of the OPRA cohort at baseline age 75, at age 80, and age 85.

	Baseline visit *n* = 963		Follow up visit 1 *n* = 683		Follow up visit 2 *n* = 355	
Characteristics	Mean	SD	Mean	SD	Mean	SD
Age (years)	75.2	0.1	80.2	0.2	85.2	0.1
Body mass index (kg/m^2^)	26.3	4.2	26.0	4.2	25.4	4.0
Weight (kg)	68	12	66	11	64	11
Height (cm)	161	6	159	6	159	6
s-FGF23 (pmol/l)	0.8^a^	0.7	1.1^a^	0.9	0.9^a^	1.1
s-25(OH)D3 (nmol/l)	62	19	73^a^	38	79	26
s-PTH^a^ (pmol/l)	4.2^a^	2.3	3.9^a^	3.4	4.2	3.2
p-hsCRP^a^ (mg/l)	1.9^a^	3.1	1.9^a^	3.4	1.7^a^	3.0
p-calcium (mmol/l)	2.41	0.07	2.41	0.13	2.34	0.09
p-albumin (g/l)	40.8	2.5	39.9	3.0	39.9	2.2
p-phosphate (mmol/l)	1.12	0.19	1.10	0.15	1.12	0.13
p-TSH (g/l)	2.36	4.1				
p-creatinine (µmol/l)	66^a^	16	71^a^	19	78^a^	22
p-Cystatin C (mg/l)	1.06^a^	0.30	1.19^a^	0.34	1.26^a^	0.37
eGFR_cys_ (ml/min/1.73 m^2^)	64.7	13.4	57.8	12.2	51.5	11.9
eGFR_crea_ (ml/min/1.73 m^2^)	66.4	11.6	59.7	12.3	51.6	12.8
eGFR_cys_/eGFR_crea_	0.98	0.19	0.98	0.15	1.01	0.18
Total body fat (kg)	26.10	7.90	25.94	8.20	24.70	8.34
Muscle strength (Nm)	269	79	246	71	221	64
Muscle mass (kg/m^2^)	6.20	0.65	5.92	0.61	5.85	0.60
Total lean mass (kg)	37. 28	3.95	36.50	3.89	36. 03	3.64
Gait speed (m/s)	1.2	0.3	1.1	0.3	1.0	0.3
	*n*	%	*n*	%	*n*	%
Sarcopenia (*n*)	32	3.3	33	4.8	36	10
SGHS (<0.6)	23	2.3	4	0.6	1	0.2
SGHS (<0.7)	64	6.6	19	3.0	8	2.2

^a^Values are presented as median (interquartile range). Muscle mass was assessed as appendicular lean mass index (appendicular lean mass [kg]/height² [m²]). eGFR was estimated using the EKFC equations based on cystatin C and creatinine.

Abbreviations: hsCRP, high-sensitivity C-reactive protein; eGFR, estimated glomerular filtration rate; eGFRcys, estimated glomerular filtration rate based on cystatin C; eGFRcrea, estimated glomerular filtration rate based on creatinine; FGF23, fibroblast growth factor 23; 25(OH)D3, 25-hydroxyvitamin D3; PTH, parathyroid hormone; THS, thyroid stimulating hormone; SD, standard deviation; SGHS, shrunken glomerular filtration syndrome; s-, serum; p-, plasma.

Sarcopenia was present in 32 (3.3%) individuals at baseline, 33 (4.8%) at ages 80 and 36 (10%) at age 85. At baseline the mean eGFR_cys_/eGFR_crea_ ratio was lower in sarcopenic than in non-sarcopenic women (0.76 vs. 0.86, *P* = .007). Similar patterns were observed at ages 80 and 85, reaching significance only at age 85 (*P* = .012), not age 80 (*P* = .178) (Table 2).

**Table 2:** eGFR_cys_/eGFR_crea_ ratio in sarcopenic^a^ ^tb2fn1^ and non-sarcopenic individuals in the OPRA cohort.

	Age 75			Age 80			Age 85		
	Sarcopenic (*n* = 32)	Non-sarcopenic (*n* = 868)	*P*-value^b^	Sarcopenic (*n* = 33)	Non-sarcopenic (*n* = 593)	*P*-value^b^	Sarcopenic (*n* = 36)	Non-sarcopenic (*n* = 298)	*P*-value^b^
eGFR ratio Mean (SD)	0.76 (±0.18)	0.86 (±0.19)	.007	0.78 (±0.16)	0.81 (±0.15)	.178	0.74 (±0.14)	0.81 (±0.16)	.012

^a^Sarcopenia defined according to the European Working Group on Sarcopenia in older people (EWGSOP)2.

^b^ *P*-value derived from independent samples *t*-test (two-tailed) comparing sarcopenic and non-sarcopenic individuals.

Scatterplots of the eGFR_cys_/eGFR_crea_ ratio in relation to FM, muscle mass, and muscle strength at baseline are shown in Fig. 3, indicating weak associations overall with considerable dispersion of data points (*R*^2^ ranging from 1.0% to 6.0%).

**Figure 3:**
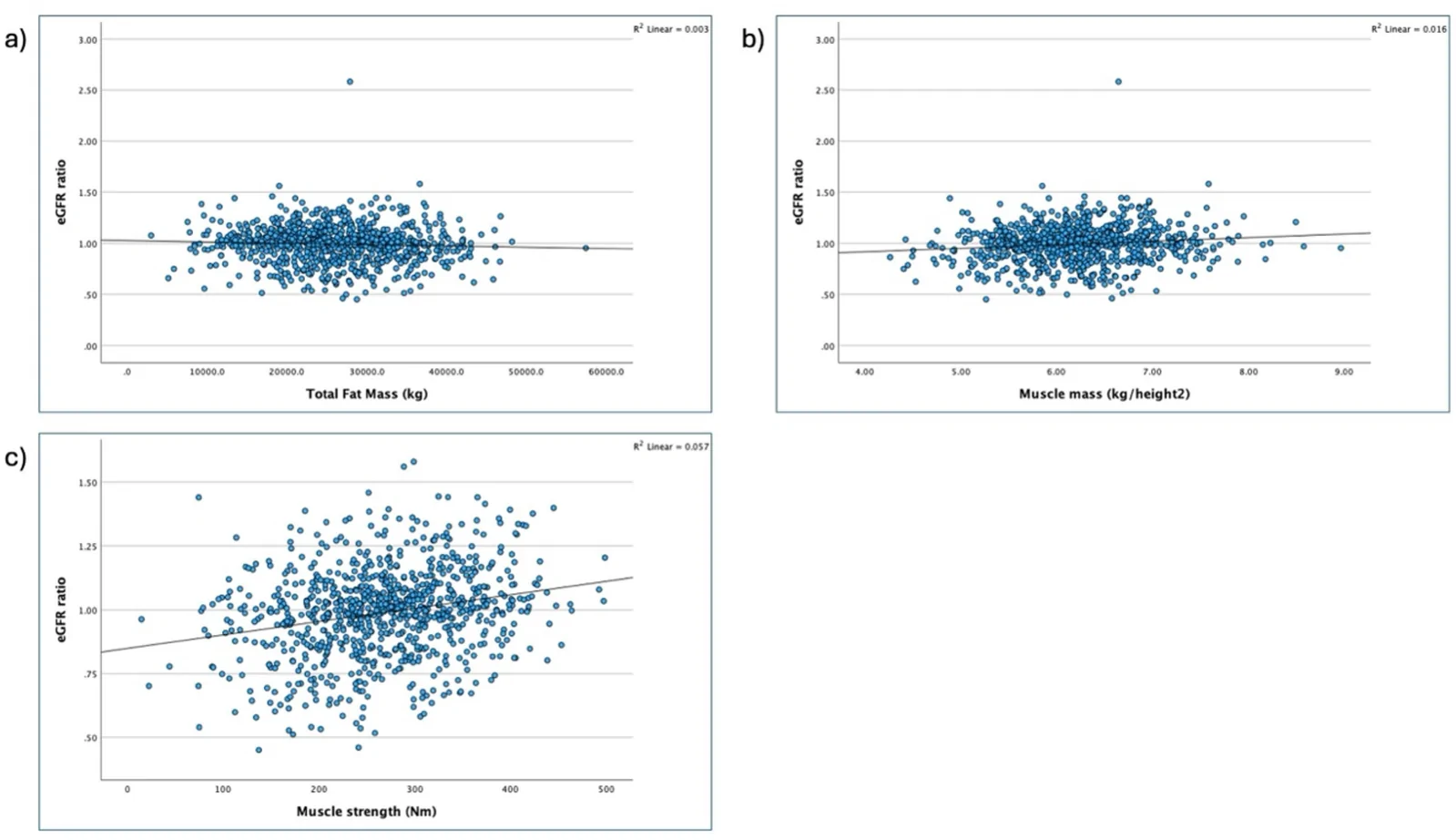
Scatterplot representing the association between the eGFR_cys_/eGFR_crea_ ratio and (a) fat mass, (b) muscle mass, and (c) muscle strength at age 75.

### Associations between the eGFR_cys_/eGFR_crea_ ratio and body composition, muscle strength, and sarcopenia

#### eGFR_cys_/eGFR_crea_ ratio and fat mass

No significant association was observed between FM and the eGFR_cys_/eGFR_crea_ ratio at ages 75, 80, and 85 in either the unadjusted or adjusted analyses (Tables 3 –5). Adjustments for smoking, hsCRP, and steroid treatment did not substantially alter the results. FM explained only a small proportion of the variation in the eGFR_cys_/eGFR_crea_ ratio (adjusted *R*^2^ values of 2.1%, 3.4%, and 0.2% at ages 75, 80, and 85, respectively). The result remained essentially unchanged after additional adjustment for TSH at baseline (*β* = −0.0015, 95% CI −0.0031 to 0.00011, *P* = .067).

**Table 3:** Linear regression of eGFR_cys_/eGFR_crea_ ratio, based on EKFC equation, and body composition (FM and muscle mass), muscle strength, and sarcopenia.

Fat mass (kg)			
Age	*β* ̂ (95% CI)	*P*-value	*R* ^2^
75 (*n* = 963)			
Unadjusted	−0.0013 (−0.0028 to 0.00026)	.104	0.2%
Model 1	−0.0011 (−0.0026 to 0.00048)	.173	2.1%
Model 2 (TSH)	−0.0015 (−0.0031 to 0.00011)	.067	3.6%
80 (*n* = 683)			
Unadjusted	−0.00078 (−0.0023 to 0.00079)	.301	0.01%
Model 1	−0.00087 (−0.0024 to 0.00063)	.256	3.4%
85 (*n* = 355)			
Unadjusted	−9.1 × 10^−5^ (−0.0024 to 0.0022)	.938	−0.3%
Model 1	0.0023 (−0.0022 to 0.0027)	.857	0.2%
Muscle mass (kg/height^2^)^a^			
Age	*β* ̂ (95% CI)	*P*-value	*R* ^2^
75 (*n* = 963)			
Unadjusted	0.035 (0.017–0.053)	<.001	1.5%
Model 1	0.034 (0.015–0.052)	<.001	3.3%
Model 2 (TSH)	0.032 (0.013–0.052)	.001	4.5%
80 (*n* = 683)			
Unadjusted	0.025 (0.006–0.045)	.009	0.9%
Model 1	0.028 (0.009–0.047)	.004	4.4%
85 (*n* = 355)			
Unadjusted	0.023 (−0.009 to 0.055)	.155	0.3%
Model 1	0.023 (−0.011 to 0.057)	.189	0.8%
Muscle strength (Nm)			
Age	*β* ̂ (95% CI)	*P*-value	*R* ^2^
75 (*n* = 963)			
Unadjusted	0.001(0.4 × 10^−3^ to 0.001)	<.001	5.6%
Model 1	0.001(0.4 × 10^−3^ to 0.001)	<.001	7.4%
Model 2 (TSH)	0.001(0.39 × 10^−3^ to 0.001)	<.001	8.8%
80 (*n* = 683)			
Unadjusted	0.46 × 10^−3^(0.28 × 10^−3^ to 0.001)	<.001	4.7%
Model 1	0.42 × 10^−3^(0.25 × 10^−3^ to 0.001)	<.001	6.0%
85 (*n* = 355)			
Unadjusted	0.001(0.2 × 10^−3^ to 0.001)	.002	3.2%
Model 1	0.001(0.1 × 10^−3^ to 0.001)	.005	2.4%
Sarcopenia			
Age	*β* ̂ (95% CI)	*P*-value	*R* ^2^
75 (*n* = 963)			
Unadjusted	−0.084 (−0.153 to −0.015)	.017	0.5%
Model 1	−0.083 (−0.151 to −0.014)	.018	3.0%
Model 2 (TSH)	−0.081 (−0.150 to −0.013)	.020	4.3%
80 (*n* = 683)			
Unadjusted	−0.051 (−0.105 to 0.003)	.065	0.4%
Model 1	−0.052 (−0.105 to 0.002)	.060	2.7%
85 (*n* = 355)			
Unadjusted	−0.068 (−0.141 to −0.012)	.021	1.3%
Model 1	−0.079 (−0.149 to −0.009)	.026	1.0%

Note Model 1 is adjusted for smoking, hsCRP, and steroid treatment. Model 2 is adjusted for TSH, hsCRP, steroid treatment and smoking at age 75.

Abbreviations: *β* ̂ , regression coefficient; CI, confidence interval; *R*^2^, adjusted partial explained variation.

^a^Muscle mass is defined as ALM-index, appendicular lean mass (kg/height^2^).

**Table 4:** Linear regression of eGFR_crea_, based on EKFC equation, and body composition (FM and muscle mass), muscle strength, and sarcopenia.

Fat mass (kg)			
Age	*β* ̂ (95% CI)	*P*-value	*R* ^2^
75 (*n* = 963)			
Unadjusted	−0.179 (−0.269 to −0.088)	<.001	1.6%
Model 1	−0.184 (−0.276 to −0.092)	<.001	1.9%
Model 2	−0.164 (−0.261 to −0.0069)	<.001	1.9%
80 (*n* = 683)			
Unadjusted	−0.308 (−0.422 to −0.196)	<.001	4.1%
Model 1	−0.307 (−0.424 to −0.190)	<.001	3.6%
85 (*n* = 355)			
Unadjusted	−0.302 (−0.406 to −0.145)	<.001	3.6%
Model 1	−0.326 (−0.493 to −0.159)	<.001	5.2%
Muscle mass (kg/height^2^)^a^			
Age	*β* ̂ (95% CI)	*P*-value	*R* ^2^
75 (*n* = 963)			
Unadjusted	−0.790 (−1.89 to 0.308)	.158	0.1%
Model 1	−0.931 (−2.04 to 0.182)	.101	0.5%
Model 2	−0.718 (−1.89 to 0.449)	.227	5.1%
80 (*n* = 683)			
Unadjusted	−1.573 (−3.084 to −0.062)	.041	0.5%
Model 1	−1.371 (−2.914 to 0.172)	.081	−0.04%
85 (*n* = 355)			
Unadjusted	−1.525 (−3.733 to 0.682)	.175	0.2%
Model 1	-1.740(-4.048 to 0.567)	.319	1.4%
Muscle strength (Nm)			
Age	*β* ̂ (95% CI)	*P*-value	*R* ^2^
75 (*n* = 963)			
Unadjusted	0.1 × 10^−3^ (−0.009 to 0.009)	.976	−0.1%
Model 1	−0.002 (−0.011 to 0.008)	.724	0.4%
Model 2	−0.001 (−0.011 to 0.008)	.775	0.9%
80 (*n* = 683)			
Unadjusted	−0.008 (−0.022 to 0.006)	.273	0.03%
Model 1	−0.006 (−0.020 to 0.009)	.445	−0.05%
85 (*n* = 355)			
Unadjusted	−0.3 × 10^−3^ (−0.023 to 0.022)	.977	−0.3%
Model 1	−0.003 (−0.028 to 0.021)	.786	1.4%
Sarcopenia			
Age	*β* ̂ (95% CI)	*P*-value	*R* ^2^
75 (*n* = 963)			
Unadjusted	−1.2 (−5.3 to 2.9)	.571	−0.1%
Model 1	−1.2 (−5.3 to 2.9)	.594	0.1%
Model 2	−1.1 (−5.3 to 2.9)	.587	0.5%
80 (*n* = 683)			
Unadjusted	3.349 (−0.900 to 7.599)	.122	0.2%
Model 1	3.995 (−0.313 to 8.304)	.069	−0.04%
85 (*n* = 355)			
Unadjusted	0.573 (−3.928 to 5.075)	.802	−0.3%
Model 1	0.057 (−4.669 to 4.782)	.981	1.6%

Note: Model 1 is adjusted for smoking, hsCRP, and steroid treatment. Model 2 is adjusted for TSH, hsCRP, steroid treatment and smoking at age 75.

Abbreviations: *β* ̂, regression coefficient; CI, confidence interval; *R*^2^, adjusted partial explained variation.

^a^Muscle mass is defined as ALM-index, appendicular lean mass (kg/height^2^).

**Table 5:** Linear regression of eGFR_cys_, based on EKFC equation, and body composition (FM and muscle mass), muscle strength, and sarcopenia.

Fat mass (kg)			
Age	*β* ̂ (95% CI)	*P*-value	*R* ^2^
75 (*n* = 963)			
Unadjusted	−0.266 (−0.374 to −0.159)	<0.001	2.5%
Model 1	−0.264 (−0.372 to −0.155)	<0.001	5.9%
Model 2 (TSH)	−0.284 (−0.397 to −0.171)	<0.001	6.3%
80 (*n* = 683)			
Unadjusted	−0.373 (−0.485 to −0.262)	<0.001	6.2%
Model 1	−0.372 (−0.486 to −0.259)	<0.001	7.1%
85 (*n* = 355)			
Unadjusted	−0.313 (−0.458 to −0.168)	<0.001	4.5%
Model 1	−0.320 (−0.474 to −0.166)	<0.001	7.3%
Muscle mass (kg/height^2^)^a^			
Age	*β* ̂ (95% CI)	*P*-value	*R* ^2^
75 (*n* = 963)			
Unadjusted	1.143 (−0.157 to 2.442)	0.085	0.2%
Model 1	0.928 (−0.388 to 2.244)	0.167	3.6%
Model 2 (TSH)	1.064 (−0.322 to 2.449)	0.132	3.7%
80 (*n* = 683)			
Unadjusted	−0.211 (−1.711 to 1.289)	0.782	−0.1%
Model 1	0.163 (−1.354 to 1.680)	0.833	0.9%
85 (*n* = 355)			
Unadjusted	−0.301 (−2.314 to 1.713)	0.769	−0.3%
Model 1	−0.748 (−2.904 to 1.407)	0.495	2.4%
Muscle strength (Nm)			
Age	*β* ̂ (95% CI)	*P*-value	*R* ^2^
75 (*n* = 963)			
Unadjusted	0.033 (0.023–0.044)	<0.001	4.1%
Model 1	0.031 (0.020–0.041)	<0.001	7.6%
Model 2 (TSH)	0.031 (0.020–0.042)	<0.001	7.5%
80 (*n* = 683)			
Unadjusted	0.019 (0.005–0.033)	0.006	1.2%
Model 1	0.020 (0.006–0.034)	0.005	1.5%
85 (*n* = 355)			
Unadjusted	0.023 (0.002–0.044)	0.030	1.3%
Model 1	0.021 (−0.002 to 0.044)	0.078	2.7%
Sarcopenia			
Age	*β* ̂ (95% CI)	*P*-value	*R* ^2^
75 (*n* = 963)			
Unadjusted	−7.510 (−12.283 to −2.736)	0.002	0.9%
Model 1	−7.339 (−12.122 to −2.557)	0.003	4.7%
Model 2 (TSH)	−7.304 (−12.117 to −2.492)	0.003	4.7%
80 (*n* = 683)			
Unadjusted	1.045 (−3.185 to 5.276)	0.628	−0.1%
Model 1	1.200 (−3.001 to 5.402)	0.575	0.5%
85 (*n* = 355)			
Unadjusted	−3.618 (−7.765 to 0.530)	0.087	0.6%
Model 1	−4.354 (−8.710 to 0.001)	0.050	3.2%

Note: Model 1 is adjusted for smoking, hsCRP, and steroid treatment. Model 2 is adjusted for TSH, hsCRP, steroid treatment and smoking at age 75.

Abbreviations: *β* ̂, regression coefficient; CI, confidence interval; *R*^2^, adjusted partial explained variation.

^a^Muscle mass is defined as ALM-index, appendicular lean mass (kg/height^2^).

#### eGFR_cys_/eGFR_crea_ ratio and muscle mass

Muscle mass was positively associated with the eGFRcys/eGFRcrea ratio at ages 75 (*P* < .001) and 80 (*P* = .004) in both unadjusted and adjusted models (Table 3), but not at age 85 (*P* = .189). Muscle mass explained only a small proportion of the variation in the eGFR_cys_/eGFR_crea_ ratio, with adjusted *R*^2^ values of 3.3% and 4.4% at ages 75 and 80, respectively.

#### eGFR_cys_/eGFR_crea_ ratio and muscle strength

Muscle strength was consistently associated with the eGFR_cys_/eGFR_crea_ ratio across all three examinations (Table 3). The association remained significant after adjustments at all age groups, although explaining only a small proportion of the variation in the ratio, with adjusted *R*^2^ values decreasing with advancing age (7.4%, 6.0%, and 2.4%).

#### eGFR_cys_/eGFR_crea_ ratio and sarcopenia

Sarcopenia, reflecting both muscle strength and mass, was inversely associated with the eGFR_cys_/eGFR_crea_ ratio at ages 75 and 85, whereas no significant association was observed at age 80 (Table 3). After adjustments for confounders, the association remained significant at ages 75 (adjusted *R*^*2*^ = −0.083, 95% CI −0.151 to −0.014, *P* = .018) and 85 (adjusted *R*^*2*^ = −0.079, 95% CI −0.149 to −0.009, *P* = .026). However, sarcopenia only accounted for a small proportion of the variation in the eGFR_cys_/eGFR_crea_ ratio, with adjusted *R*^2^ values of 3.0% and 1.0% at ages 75 and 85, respectively.

### Sensitivity analyses

Mean eGFR values calculated using the LM-rev, CAPA, and CKD-EPI equations are presented in Table S1. Across all equations eGFR decreased with increasing age, with equation-specific differences consistent with previous reports [12].

In the sensitivity analyses using the CAPA and LM-rev equations (Table S2a), the associations with muscle mass, muscle strength, and sarcopenia were consistent with the primary EKFC-based analysis in both direction and statistical significance, with similar effect estimates (e.g. muscle strength at age 75: *β*=0.001, 95% CI 0.0005–0.001, *P* < .001). In contrast to the primary analysis, FM was significantly and inversely associated with the ratio at ages 75 (*β* = −0.0032, 95% CI −0.0051 to −0.0013, *P* < .001) and 80 (*β* = −0.0024, 95% CI −0.0040 to −0.00085, *P* = .002) using the CAPA/LM-rev equations, an association not observed with the EKFC-based ratio (Table 3); this discrepancy was not present at age 85 (*P* = .252). Sensitivity analyses using the CKD-EPI equations (Table S3a) showed the same pattern: muscle mass, muscle strength, and sarcopenia remained consistent with the primary analysis, while FM was again significantly and inversely associated with the ratio at ages 75 (*β* = −0.0025, 95% CI −0.0041 to −0.00082, *P* = .003) and 80 (*β* = −0.0022, 95% CI −0.0036 to −0.00079, *P* = .002) but not at age 85 (*P* = .399). These associations remained materially unchanged after additional adjustment for TSH.

### Associations with eGFR_crea_ and eGFR_cys_ individually

In separate regression analyses using eGFR_crea_ and eGFR_cys_ individually as dependent variables (Tables 4 and 5), FM was inversely associated with both eGFR_crea_ and eGFR_cys_ at all ages (*P* < .001), including after adjustment for TSH at age 75. In contrast, FM was not associated with the eGFR_cys_/eGFR_crea_ ratio (Table 3). Muscle strength was positively associated with eGFR_cys_ at ages 75 (*P* = .005) and 80 (*P* = .006) and in unadjusted analysis at age 85 (*P* = .030) but was not associated with eGFR_crea_ at any age. Muscle mass was not significantly associated with either eGFR_crea_ or eGFR_cys_ individually. Sarcopenia was associated with lower eGFR_cys_ at age 75 (*P* = .003) and borderline at age 85 (*P* = .050) but was not associated with eGFR_crea_ at any age.

## DISCUSSION

We investigated the association between the eGFR_cys_/eGFR_crea_ ratio and non-GFR determinants, including body composition, muscle strength, and sarcopenia, in older women. Overall, all the examined factors demonstrated weak or absent associations with the ratio and explained only a small proportion of its variability, suggesting that factors beyond body composition and muscular determinants contribute more substantially to the eGFR_cys_/eGFR_crea_ ratio in aging women.

### Intrinsic renal factors

Our findings support the hypothesis that intrinsic renal mechanisms, rather than non-renal factors, are important determinants of the eGFR_cys_/eGFR_crea_ ratio in older women. The limited explanatory power of body composition-related variables is consistent with the concept of selective glomerular hypofiltration, whereby reduced clearance of larger molecules such as cystatin C may contribute to a lower eGFR_cys_/eGFR_crea_ ratio [13]. Age-related structural changes in the kidneys may further contribute to these findings. Aging is associated with nephron loss, glomerulosclerosis, and alterations of the filtration barrier, including thickening of the glomerular basement membrane, mesangial expansion, and reduced podocyte density accompanied by compensatory hypertrophy and hyperfiltration in the remaining nephrons [39, 40]. Importantly, such changes may preserve overall GFR at the single-nephron level while altering the properties of the filtration barrier [41]. Such changes may preferentially increase the diffusion distances and modify the permeability of larger solutes [42]. Lifetime exposure to estrogen has been suggested to exert positive effects on renal structure and function, potentially contributing sex-related differences in renal aging [43]. Collectively, these mechanisms provide a biologically plausible explanation for reduced eGFR_cys_/eGFR_crea_ ratio in older individuals, independent of non-GFR determinants. In the OPRA cohort, 2%–6% fulfilled SGHS criteria using a cutoff ratio <0.7 (Table 1).

### Non-renal factors

Body composition has been proposed as an alternative non-renal explanation for discrepancies between eGFR_cys_ and eGFR_crea_. However, its contribution appeared limited in the present study. A low plasma creatinine may reflect sarcopenia or poor nutritional status rather than preserved kidney function in ageing populations [44]. Although muscle mass was positively associated with the eGFR_cys_/eGFR_crea_ ratio at ages 75 and 80, it explained <5% of the observed variation, despite the established relationship between muscle mass and creatinine concentrations, particularly in younger populations [26, 45, 46]. Additional age- and sex-specific patterns have been reported, including declining creatinine in aging men but stable levels in women [47]. This observation suggests that age-related reductions in muscle mass can lower creatinine concentrations and lead to inflated eGFR, complicating the assessment of kidney function in older adults.

Sarcopenia was associated with a lower eGFR_cys_/eGFR_crea_ ratio at baseline, suggesting that extreme variants of body composition may partly contribute to discrepancies between eGFR_cys_ and eGFR_crea_. However, only 3% of participants met the strict EWGSOP2 criteria for sarcopenia, reflecting this healthy, community-dwelling cohort, and limiting generalizability to frailer populations.

Muscle strength remained associated with the ratio across all three time points but with small, decreasing effect sizes with age, possibly reflecting that muscle strength better captures functional decline than muscle mass as frailty progresses [6, 38, 48]. In contrast to previous reports linking adiposity to higher cystatin C concentrations through adipose-tissue-related metabolic and inflammatory pathways, FM was not associated with the ratio at any age in the present study [19, 49], further supporting the notion that adipose tissue contributes minimally to variation in the ratio among older women. In the sensitivity analysis using the CAPA and LM-rev equations, in contrast, FM was significantly and inversely associated with the ratio at ages 75 and 80, an association not observed with the primary EKFC-based ratio; this equation-dependent finding suggests that FM may still contribute modestly to the ratio through mechanisms not fully captured by the EKFC equation, although it did not alter our main conclusions [19, 49, 50]. Taken together, these results suggest that the influence of muscular and other body composition-related factors on the eGFR_cys_/eGFR_crea_ ratio diminishes with age, while non-muscular determinants may become increasingly important. The absence of significant associations at age 85 may reflect both a true attenuation of these relationships with advancing age and reduced statistical power due to the smaller sample size at the oldest follow-up.

### Strengths and limitations

To our knowledge, this is the first study to examine the association between the eGFR_cys_/eGFR_crea_ ratio and detailed measures of body composition, muscle strength, and sarcopenia in women. Strengths include the well-characterized, age-homogenous OPRA cohort with a high participation rate (65%), reducing selection bias and confounding by sex and age; DXA-derived body composition measurements and objective muscle strength assessments; and repeated measurements over a 10-year follow-up.

Several limitations should be acknowledged. Although the restricted study population enhances internal validity, it may limit generalizability to other populations. Furthermore, as the women in this cohort had largely preserved kidney function at baseline (Table 1), the findings may not be generalizable to individuals with moderate to advanced CKD. In addition, residual confounding such as dietary protein intake, detailed information about thyroid disease, polypharmacy, and comorbidities cannot be excluded. Finally, direct measurements of glomerular filtration rate were not available, which would have strengthened the interpretation of the ratio.

## CONCLUSION

In conclusion, body composition, muscle strength, and sarcopenia were only weakly associated with the eGFR_cys_/eGFR_crea_ ratio in this cohort of older women. These findings suggest that non-renal factors related to body composition are unlikely to fully account for a low eGFR_cys_/eGFR_crea_ ratio, supporting the possibility that intrinsic renal mechanisms, such as SGHS, may play a prominent role. Further studies are needed to clarify the determinants and clinical implications of the eGFR_cys_/eGFR_crea_ ratio in diverse populations.

## Supplementary Material

sfag379_Supplemental_Files

## Data Availability

The data underlying this article will be shared on reasonable request to the corresponding author.

## References

[1] Mark PB, Stafford LK, Grams ME et al. Global, regional, and national burden of chronic kidney disease in adults, 1990–2023, and its attributable risk factors: a systematic analysis for the Global Burden of Disease Study 2023. Lancet. 2025;406:2461–82. 10.1016/S0140-6736(25)01853-7 41213283

[2] Go AS, Chertow GM, Fan D et al. Chronic kidney disease and the risks of death, cardiovascular events, and hospitalization. N Engl J Med. 2004;351:1296–305. 10.1056/NEJMoa041031 15385656

[3] Inker LA, Schmid CH, Tighiouart H et al. Estimating glomerular filtration rate from serum creatinine and cystatin C. N Engl J Med. 2012;367:20–9. 10.1056/NEJMoa1114248 PMC4398023 22762315

[4] Soveri I, Berg UB, Björk J et al. Measuring GFR: a systematic review. Am J Kidney Dis. 2014;64:411–24. 10.1053/j.ajkd.2014.04.010 24840668

[5] Dharnidharka VR, Kwon C, Stevens G. Serum cystatin C is superior to serum creatinine as a marker of kidney function: a meta-analysis. Am J Kidney Dis. 2002;40:221–6. 10.1053/ajkd.2002.34487 12148093

[6] Shlipak MG, Matsushita K, Ärnlöv J et al. Cystatin C versus creatinine in determining risk based on kidney function. N Engl J Med. 2013;369:932–43. 10.1056/NEJMoa1214234 PMC3993094 24004120

[7] Chen J, Budoff MJ, Reilly MP et al. Coronary artery calcification and risk of cardiovascular disease and death among patients with chronic kidney disease. JAMA Cardiol. 2017;2:635. 10.1001/jamacardio.2017.0363 PMC5798875 28329057

[8] Willey JZ, Moon YP, Husain SA et al. Creatinine versus cystatin C for renal function-based mortality prediction in an elderly cohort: the Northern Manhattan Study. PLoS One. 2020;15:e0226509. 10.1371/journal.pone.0226509 PMC6961921 31940363

[9] Zhang L, Luo M, Xie X et al. Shrunken pore syndrome: a new and more powerful phenotype of renal dysfunction than chronic kidney disease for predicting contrast-associated acute kidney injury. JAHA. 2023;12:e027980. 10.1161/JAHA.122.027980 PMC9973563 36565177

[10] Åkesson A, Lindström V, Nyman U et al. Shrunken pore syndrome and mortality: a cohort study of patients with measured GFR and known comorbidities. Scand J Clin Lab Invest. 2020;80:412–22. 10.1080/00365513.2020.1759139 32459111

[11] Purde MT, Nock S, Risch L et al. Ratio of cystatin C and creatinine-based estimates of the glomerular filtration rate predicts mortality in healthy seniors independent of kidney function. Scand J Clin Lab Invest. 2016;76:341–3. 10.3109/00365513.2016.1149882 26981764

[12] Malmgren L, McGuigan FE, Christensson A et al. Impaired selective renal filtration captured by eGFR_cysC_/eGFR_crea_ ratio is associated with mortality in a population based cohort of older women. Sci Rep. 2022;12:1273. 10.1038/s41598-022-05320-w PMC8786879 35075286

[13] Grubb A, Lindström V, Jonsson M et al. Reduction in glomerular pore size is not restricted to pregnant women. Evidence for a new syndrome: ‘Shrunken pore syndrome’. Scand J Clin Lab Invest. 2015;75:333–40. 10.3109/00365513.2015.1025427 PMC4487590 25919022

[14] Ohlsson MA, Nilsson C, Molvin J et al. Association of Osteopontin and selective glomerular hypofiltration syndrome with hospitalization for kidney failure in acute heart failure patients. BMC Nephrol. 2025;26:550. 10.1186/s12882-025-04481-w PMC12502603 41053620

[15] Grubb A. Shrunken pore syndrome—a common kidney disorder with high mortality. Diagnosis, prevalence, pathophysiology and treatment options. Clin Biochem. 2020;83:12–20. 10.1016/j.clinbiochem.2020.06.002 32544475

[16] Xhakollari L, Grubb A, Jujic A et al. The Shrunken pore syndrome is associated with poor prognosis and lower quality of life in heart failure patients: the HARVEST-Malmö study. ESC Heart Fail. 2021;8:3577–86. 10.1002/ehf2.13485 PMC8497365 34382359

[17] Malmgren L, Öberg C, den Bakker E et al. The complexity of kidney disease and diagnosing it—cystatin C, selective glomerular hypofiltration syndromes and proteome regulation. J Intern Med. 2023;293:293–308. 10.1111/joim.13589 PMC10107454 36385445

[18] Dardashti A, Nozohoor S, Grubb A et al. Shrunken pore syndrome is associated with a sharp rise in mortality in patients undergoing elective coronary artery bypass grafting. Scand J Clin Lab Invest. 2016;76:74–81. 10.3109/00365513.2015.1099724 PMC4720044 26647957

[19] Katz-Agranov N, Rieu-Werden ML, Thacker A et al. Large discordance between creatinine-based and cystatin C–based eGFRs is associated with falls, hospitalizations, and death in older adults. Clin J Am Soc Nephrol. 2024;19:1275–83. 10.2215/CJN.0000000000000523 PMC11469786 39146034

[20] Estrella MM, Ballew SH, Sang Y et al. Discordance in creatinine- and cystatin C–based eGFR and clinical outcomes. JAMA. 2025;334:1915. 10.1001/jama.2025.17578 PMC12595547 41202182

[21] Farrington DK, Surapaneni A, Matsushita K et al. Discrepancies between cystatin C-based and creatinine-based eGFR. Clin J Am Soc Nephrol. 2023;18:1143–52. 10.2215/CJN.0000000000000217 PMC10564370 37339177

[22] Cong X, Chen X, Shen Q et al. Serum cystatin C levels increase with increasing visceral fat area in patients with type 2 diabetes mellitus. Sci Rep. 2024;14:18638. 10.1038/s41598-024-69623-w PMC11317477 39128944

[23] Laucyte-Cibulskiene A, Nilsson PM, Engström G et al. Increased fat mass index is associated with decreased glomerular filtration rate estimated from cystatin C. Data from Malmö Diet and Cancer cohort. PLoS One. 2022;17:e0271638. 10.1371/journal.pone.0271638 PMC9302820 35862349

[24] Baxmann ACAA, Ahmed MS, Marques NAAC et al. Influence of muscle mass and physical activity on serum and urinary creatinine and serum cystatin C. Clin J Am Soc Nephrol. 2008;3:348–54. 10.2215/CJN.02870707 PMC2390952 18235143

[25] Fricker M, Wiesli P, Brändle M et al. Impact of thyroid dysfunction on serum cystatin C. Kidney Int. 2003;63:1944–7. 10.1046/j.1523-1755.2003.00925.x 12675875

[26] Fu EL, Levey AS, Coresh J et al. Accuracy of GFR estimating equations in patients with discordances between creatinine and cystatin C-based estimations. JASN. 2023;34:1241–51. 10.1681/ASN.0000000000000128 PMC10356168 36995139

[27] Bartholomae E, Knurick J, Johnston CS. Serum creatinine as an indicator of lean body mass in vegetarians and omnivores. Front Nutr. 2022;9:996541. 10.3389/fnut.2022.996541 PMC9525150 36185683

[28] Groothof D, Shehab NBN, Erler NS et al. Creatinine, cystatin C, muscle mass, and mortality: findings from a primary and replication population-based cohort. J Cachexia Sarcopenia Muscle. 2024;15:1528–38. 10.1002/jcsm.13511 PMC11294032 38898741

[29] Beaudart C, Alcazar J, Aprahamian I et al. Health outcomes of sarcopenia: a consensus report by the outcome working group of the Global Leadership Initiative in Sarcopenia (GLIS). Aging Clin Exp Res. 2025;37:100. 10.1007/s40520-025-02995-9 PMC11929733 40120052

[30] Yeung SSY, Reijnierse EM, Pham VK et al. Sarcopenia and its association with falls and fractures in older adults: a systematic review and meta-analysis. J Cachexia Sarcopenia Muscle. 2019;10:485–500. 10.1002/jcsm.12411 PMC6596401 30993881

[31] Aversa Z, Zhang X, Fielding RA et al. The clinical impact and biological mechanisms of skeletal muscle aging. Bone. 2019;127:26–36. 10.1016/j.bone.2019.05.021 PMC6708726 31128290

[32] Jackson AS, Janssen I, Sui X et al. Longitudinal changes in body composition associated with healthy ageing: men, aged 20–96 years. Br J Nutr. 2012;107:1085–91. 10.1017/S0007114511003886 21810289

[33] Tay L, Ding YY, Leung BP et al. Sex-specific differences in risk factors for sarcopenia amongst community-dwelling older adults. Age (Dordr). 2015;37:121. 10.1007/s11357-015-9860-3 PMC5005859 26607157

[34] Gerdhem P, Ivaska KK, Alatalo SL et al. Biochemical markers of bone metabolism and prediction of fracture in elderly women. J Bone Miner Res. 2004;19:386–93. 10.1359/JBMR.0301244 15040826

[35] Berglundh S, Malmgren L, Luthman H et al. C-reactive protein, bone loss, fracture, and mortality in elderly women: a longitudinal study in the OPRA cohort. Osteoporos Int. 2015;26:727–35. 10.1007/s00198-014-2951-7 25410434

[36] Pottel H, Delanaye P. Development and validation of a modified full age spectrum creatinine-based equation to estimate glomerular filtration rate. Ann Intern Med. 2021;174:1038. 10.7326/L21-0248 34280339

[37] Pottel H, Björk J, Rule AD et al. Cystatin C–based equation to estimate GFR without the inclusion of race and sex. N Engl J Med. 2023;388:333–43. 10.1056/NEJMoa2203769 36720134

[38] Heymsfield SB, Gonzalez MC, Lu J et al. Skeletal muscle mass and quality: evolution of modern measurement concepts in the context of sarcopenia. Proc Nutr Soc. 2015;74:355–66. 10.1017/S0029665115000129 25851205

[39] Cruz-Jentoft AJ, Bahat G, Bauer J et al. Sarcopenia: revised European consensus on definition and diagnosis. Age Ageing. 2019;48:16–31. 10.1093/ageing/afy169 PMC6322506 30312372

[40] Denic A, Lieske JC, Chakkera HA et al. The substantial loss of nephrons in healthy human kidneys with aging. JASN. 2017;28:313–20. 10.1681/ASN.2016020154 PMC5198286 27401688

[41] Fang Y, Gong AY, Haller ST et al. The ageing kidney: molecular mechanisms and clinical implications. Ageing Res Rev. 2020;63:101151. 10.1016/j.arr.2020.101151 PMC7595250 32835891

[42] Hommos MS, Glassock RJ, Rule AD. Structural and functional changes in human kidneys with healthy aging. JASN. 2017;28:2838–44. 10.1681/ASN.2017040421 PMC5619977 28790143

[43] Eckersley A, Morais MR, Ozols M et al. Peptide location fingerprinting identifies structural alterations within basement membrane components in ageing kidney. Matrix Biol. 2023;121:167–78. 10.1016/j.matbio.2023.07.001 PMC7619537 37437747

[44] Bouderlique E, Pszczolinski R, Prot-Bertoye C et al. Glomerular filtration rate and sexual dimorphism: lessons from animal and human studies. Curr Opin Nephrol Hypertens. 2025;34:330–5. 10.1097/MNH.0000000000001079 40265514

[45] Thongprayoon C, Cheungpasitporn W, Kashani K. Serum creatinine level, a surrogate of muscle mass, predicts mortality in critically ill patients. J Thorac Dis. 2016;8:E305–E11. 10.21037/jtd.2016.03.62 PMC4842835 27162688

[46] Groothof D, Post A, Polinder-Bos HA et al. Muscle mass and estimates of renal function: a longitudinal cohort study. J Cachexia Sarcopenia Muscle. 2022;13:2031–43. 10.1002/jcsm.12969 PMC9398222 35596604

[47] Nankivell BJ, Nankivell LFJ, Elder GJ et al. How unmeasured muscle mass affects estimated GFR and diagnostic inaccuracy. EClinicalMedicine. 2020;29-30:100662. 10.1016/j.eclinm.2020.100662 PMC7788434 33437955

[48] Yim J, Son NH, Kyong T et al. Muscle mass has a greater impact on serum creatinine levels in older males than in females. Heliyon. 2023;9:e21866. 10.1016/j.heliyon.2023.e21866 PMC10663898 38027624

[49] Björk J, Grubb A, Larsson A et al. Accuracy of GFR estimating equations combining standardized cystatin C and creatinine assays: a cross-sectional study in Sweden. Clin Chem Lab Med. 2015;53:403–14. 10.1515/cclm-2014-0578 25274955

[50] Mueller-Peltzer K, von Krüchten R, Lorbeer R et al. Adipose tissue is associated with kidney function parameters. Sci Rep. 2023;13:9151. 10.1038/s41598-023-36390-z PMC10244418 37280396

