# Supplementary material for: Body composition has limited influence on the eGFRcys/eGFRcrea ratio in older women
Source: Clin Kidney J. 2026 Oct 28;19(11):sfag379. doi: 10.1093/ckj/sfag379 (PMC13748965; doi:10.1093/ckj/sfag379)
Supplement: Supplementary file 1 [file sfag379_supplemental_files.zip › Supplementary Figure 1. .docx]

***Supplementary Figure 1.*** Directed acyclical graph identifying confounders.
