# Supplementary material for: Body composition has limited influence on the eGFRcys/eGFRcrea ratio in older women
Source: Clin Kidney J. 2026 Oct 28;19(11):sfag379. doi: 10.1093/ckj/sfag379 (PMC13748965; doi:10.1093/ckj/sfag379)
Supplement: Supplementary file 1 [file sfag379_supplemental_files.zip › Supplementary Table 1.docx]

***Supplementary Table 1****.* eGFR (mL/min/1.73m^2^) based on EKFC, CAPA, LM-rev and CKD-EPI formulas in the OPRA cohort.

|  | Age 75  n=963 | | Age 80  n=683 | | Age 85  n=355 | |
| --- | --- | --- | --- | --- | --- | --- |
| *eGFR (mL/min/1.73m^2^)* | *Mean* | *(SD)* | *Mean* | *(SD)* | *Mean* | *(SD)* |
| eGFR_crea_ (EKFC) | 66.4 | 11.6 | 59.7 | 12.3 | 51.6 | 12.8 |
|  |  |  |  |  |  |  |
| eGFR_cys_ (EKFC) | 64.7 | 13.4 | 57.8 | 12.2 | 51.5 | 11.9 |
|  |  |  |  |  |  |  |
| eGFR_crea_ (LM-rev) | 66.2 | 10.9 | 59.9 | 11.6 | 52.2 | 12.1 |
|  |  |  |  |  |  |  |
| eGFR_cys_ (CAPA) | 66.8 | 18.1 | 58.7 | 14.6 | 52.3 | 13.9 |
|  |  |  |  |  |  |  |
| eGFR_crea_ (CKD-EPI) | 74.7 | 13.4 | 68.2 | 14.4 | 59.6 | 15.1 |
|  |  |  |  |  |  |  |
| eGFR_cys_ (CKD-EPI) | 63.3 | 17.7 | 54.5 | 14.8 | 47.5 | 13.6 |
