# Supplementary material for: Body composition has limited influence on the eGFRcys/eGFRcrea ratio in older women
Source: Clin Kidney J. 2026 Oct 28;19(11):sfag379. doi: 10.1093/ckj/sfag379 (PMC13748965; doi:10.1093/ckj/sfag379)
Supplement: Supplementary file 1 [file sfag379_supplemental_files.zip › Supplementary Table 2 (LM.CAPA).docx]

***Supplementary Table 2a.*** Sensitivity analysis using CAPA and LM-rev formulas to calculate the eGFR ratio. Linear regression of eGFRcys/eGFR_crea_ ratio and body composition (fat mass and muscle mass), muscle strength and sarcopenia.

| *Fat mass (kg)* | | | |
| --- | --- | --- | --- |
| *Age* | *𝛃 ̂ (95% CI)* | *p-value* | *R^2^* |
| 75 (n=963) |  | | |
| Unadjusted | -0.0032(-0.0051 to -0.0013) | <0.001 | 1.2% |
| Model 1 | -0.0034(-0.0048 to -0.0011) | 0.002 | 4.0% |
| Model 2 | -0.0034(-0.0053 to -0.0014) | <0.001 | 5.2% |
| 80 (n=683) |  | | |
| Unadjusted | -0.0024(-0.0040 to -0.00085) | 0.002 | 1.3% |
| Model 1 | -0.0025(-0.0041 to -0.0010) | 0.001 | 5.9% |
| 85 (n=355) |  | |  |
| Unadjusted | -0.0014(-0.0037 to 0.0010) | 0.252 | 0.1% |
| Model 1 | -0.0012(-0.0037 to 0.0013) | 0.354 | 1.0% |
| *Muscle mass (kg/height^2^)** | | | |
| *Age* | *𝛃 ̂ (95% CI)* | *p-value* | *R^2^* |
| 75 (n=963) |  | | |
| Unadjusted | 0.036(0.013 to 0.059) | 0.002 | 1.0% |
| Model 1 | 0.034(0.011 to 0.056) | 0.004 | 3.9% |
| Model 2 | 0.035(0.011 to 0.058) | 0.004 | 4.8% |
| 80 (n=683) |  | | |
| Unadjusted | 0.022(0.001 to 0.042) | 0.037 | 0.5% |
| Model 1 | 0.025(0.005 to 0.046) | 0.014 | 5.2% |
| 85 (n=355) |  | |  |
| Unadjusted | 0.021(-0.011 to 0.054) | 0.202 | 0.2% |
| Model 1 | 0.019(-0.015 to 0.053) | 0.272 | 1.1% |
| *Muscle strength (Nm)* | | | |
| *Age* | *𝛃 ̂ (95% CI)* | *p-value* | *R^2^* |
| 75 (n=963) |  | | |
| Unadjusted | 0.001(0.5 x10^-3^ to 0.001) | <0.001 | 5.9% |
| Model 1 | 0.001(0.4 x10^-3^ to 0.001) | <0.001 | 8.6% |
| Model 2 | 0.001(0.4 x10^-3^ to 0.001) | <0.001 | 9.9% |
| 80 (n=683) |  |  |  |
| Unadjusted | 0.5 x10^-3^(0.3 x10^-3^ to 0.001) | <0.001 | 4.8% |
| Model 1 | 0.4 x10^-3^(0.3 x10^-3^ to 0.001) | <0.001 | 7.2% |
| 85 (n=355) |  |  |  |
| Unadjusted | 0.001(0.2 x10^-3^ to 0.001) | 0.001 | 3.3% |
| Model 1 | 0.001(0.2 x10^-3^ to 0.001) | 0.004 | 2.3% |
| *Sarcopenia* | | | |
| *Age* | *𝛃 ̂ (95% CI)* | *p-value* | *R^2^* |
| 75 (n=963) |  | | |
| Unadjusted | -0.12 (-0.21 to -0.038) | 0.004 | 0.8% |
| Model 1 | -0.12 (-0.20 to -0.038) | 0.004 | 4.3% |
| Model 2 | -0.12 (-0.20 to -0.036) | 0.005 | 5.2% |
| 80 (n=683) |  | | |
| Unadjusted | -0.04 (-0.1 to 0.02) | 0.149 | 0.2% |
| Model 1 | -0.04 (-0.1 to 0.02) | 0.147 | 3.5% |
| 85 (n=355) |  | | |
| Unadjusted | -0.08 (-0.14 to -0.012) | 0.020 | 1.3% |
| Model 1 | -0.08 (-0.15 to -0.012) | 0.012 | 1.3% |

*Model 1 is adjusted for smoking, hsCRP and steroid treatment.*

*Model 2 is adjusted for TSH, hsCRP, steroid treatment and smoking at age 75*.

*𝛃 ̂ = regression coefficient*

*CI = confidence interval*

*R^2^=adjusted partial explained variation
*=Muscle mass is defined as ALM-index, appendicular lean mass(kg/height^2^).*

***Supplementary Table 2b.*** Sensitivity analysis using revised Lund-Malmö formula to calculate eGFR based on creatinine. Linear regression of eGFR_crea_ and body composition (fat mass and muscle mass), muscle strength and sarcopenia.

| *Fat mass (kg)* | | | |
| --- | --- | --- | --- |
| *Age* | *𝛃 ̂ (95% CI)* | *p-value* | *R^2^* |
| 75 (n=963) |  | | |
| Unadjusted | -0.16(-0.24 to -0.08) | <0.001 | 1.4% |
| Model 1 | -0.16(-0.25 to -0.08) | <0.001 | 1.7% |
| Model 2 | -0.15(-0.24 to -0.06) | 0.001 | 1.8% |
| 80 (n=683) |  | | |
| Unadjusted | -0.28(-0.39 to -0.18) | <0.001 | 4.0% |
| Model 1 | -0.28(-0.39 to -0.17) | <0.001 | 3.6% |
| 85 (n=355) |  | |  |
| Unadjusted | -0.27(-0.42 to -0.12) | <0.001 | 3.2% |
| Model 1 | -0.29(-0.45 to -0.14) | <0.001 | 5.0% |
| *Muscle mass (kg/height^2^)** | | | |
| *Age* | *𝛃 ̂ (95% CI)* | *p-value* | *R^2^* |
| 75 (n=963) |  | | |
| Unadjusted | -0.763(-1.790 to 0.264) | 0.145 | 0.1% |
| Model 1 | -0.901(-1.943 to 0.141) | 0.090 | 0.5% |
| Model 2 | -0.723(-1.820 to 0.374) | 0.196 | 0.7% |
| 80 (n=683) |  | | |
| Unadjusted | -1.440 (-2.849 to -0.031) | 0.045 | 0.5% |
| Model 1 | -1.247(-2.684 to 0.190) | 0.089 | -0.005% |
| 85 (n=355) |  | |  |
| Unadjusted | -1.400(-3.484 to 0.84) | 0.187 | 0.2% |
| Model 1 | -1.647(-3.826 to 0.532) | 0.138 | 1.5% |
| *Muscle strength (Nm)* | | | |
| *Age* | *𝛃 ̂ (95% CI)* | *p-value* | *R^2^* |
| 75 (n=963) |  | | |
| Unadjusted | -0.001(-0.009 to 0.007) | 0.823 | -0.1% |
| Model 1 | -0.003(-0.011 to 0.006) | 0.549 | 0.4% |
| Model 2 | -0.002(-0.011 to 0.006) | 0.583 | 0.9% |
| 80 (n=683) |  |  |  |
| Unadjusted | -0.006(-0.019 to 0.006) | 0.326 | -0.006% |
| Model 1 | -0.004(-0.017 to 0.009) | 0.540 | -0.4% |
| 85 (n=355) |  |  |  |
| Unadjusted | 0.001(-0.021 to 0.022) | 0.941 | -0.3% |
| Model 1 | -0.002(-0.026 to 0.021) | 0.852 | 1.7% |
| *Sarcopenia* | | | |
| *Age* | *𝛃 ̂ (95% CI)* | *p-value* | *R^2^* |
| 75 (n=963) |  | | |
| Unadjusted | -1.174(-4.996 to 2.648) | 0.547 | -0.1% |
| Model 1 | -1.104(-4.939 to 2.731) | 0.572 | 0.01% |
| Model 2 | -1.137(-4.995 to 2.721) | 0.563 | 0.4% |
| 80 (n=683) |  | | |
| Unadjusted | 3.412(-0.552 to 7.377) | 0.091 | 0.3% |
| Model 1 | 3.972(-0.041 to 7.985) | 0.052 | 0.1% |
| 85 (n=355) |  | | |
| Unadjusted | 0.140(-4.099 to 4.378) | 0.948 | -0.3% |
| Model 1 | -0.300(-4.751 to 4.152) | 0.895 | 1.9% |

*Model 1 is adjusted for smoking, hsCRP and steroid treatment.*

*Model 2 is adjusted for TSH, hsCRP, steroid treatment and smoking at age 75*.

*𝛃 ̂ = regression coefficient*

*CI = confidence interval*

*R^2^=adjusted partial explained variation
*=Muscle mass is defined as ALM-index, appendicular lean mass(kg/height^2^).*

***Supplementary Table 2c.*** Sensitivity analysis using CAPA formula to calculate the eGFR based on cystatin C. Linear regression of eGFR_cys_ and body composition (fat mass and muscle mass), muscle strength and sarcopenia.

| *Fat mass (kg)* | | | |
| --- | --- | --- | --- |
| *Age* | *𝛃 ̂ (95% CI)* | *p-value* | *R^2^* |
| 75 (n=963) |  | | |
| Unadjusted | -0.38(-1.0 to -0.24) | <0.001 | 2.8% |
| Model 1 | -0.38(-1.0 to -0.23) | <0.001 | 5.8% |
| Model 2 | -0.39(-1.0 to -0.24) | <0.001 | 5.9% |
| 80 (n=683) |  | | |
| Unadjusted | -0.44(-1.0 to -0.31) | <0.001 | 6.1% |
| Model 1 | -0.44(-1.0 to -0.31) | <0.001 | 7.3% |
| 85 (n=355) |  | |  |
| Unadjusted | -0.35(-1.0 to -0.18) | <0.001 | 4.2% |
| Model 1 | -0.37(-1.0 to -0.19) | <0.001 | 6.9% |
| *Muscle mass (kg/height^2^)** | | | |
| *Age* | *𝛃 ̂ (95% CI)* | *p-value* | *R^2^* |
| 75 (n=963) |  | | |
| Unadjusted | 1.068(-0.717 to 2.854) | 0.241 | 0.04% |
| Model 1 | 0.776(-1.036 to 2.588) | 0.401 | 3.1% |
| Model 2 | 1.023(-0.881 to 2.927) | 0.292 | 3.1% |
| 80 (n=683) |  | | |
| Unadjusted | -0.263(-2.057 to 1.531) | 0.774 | -0.1% |
| Model 1 | 0.146(-1.667 to 1.959) | 0.875 | 1.1% |
| 85 (n=355) |  | |  |
| Unadjusted | -0.284(-2.619 to 2.051) | 0.811 | -0.3% |
| Model 1 | -0.850(-3.341 to 1.641) | 0.502 | 2.1% |
| *Muscle strength (Nm)* | | | |
| *Age* | *𝛃 ̂ (95% CI)* | *p-value* | *R^2^* |
| 75 (n=963) |  | | |
| Unadjusted | 0.039(0.025 to 0.053) | <0.001 | 3.1% |
| Model 1 | 0.036(0.021 to 0.050) | <0.001 | 6.1% |
| Model 2 | 0.036(0.022 to 0.051) | <0.001 | 6.1% |
| 80 (n=683) |  |  |  |
| Unadjusted | 0.023(0.007 to 0.040) | 0.006 | 1.2% |
| Model 1 | 0.024(0.007 to 0.041) | 0.005 | 1.5% |
| 85 (n=355) |  |  |  |
| Unadjusted | 0.025(0.0005 to 0.050) | 0.046 | 1.0% |
| Model 1 | 0.021(-0.006 to 0.048) | 0.120 | 2.1% |
| *Sarcopenia* | | | |
| *Age* | *𝛃 ̂ (95% CI)* | *p-value* | *R^2^* |
| 75 (n=963) |  | | |
| Unadjusted | -9.136(-15.696 to -2.577) | 0.006 | 0.7% |
| Model 1 | -8.919(-15.514 to -2.324) | 0.008 | 4.0% |
| Model 2 | -8.870(-15.494 to -2.245) | 0.009 | 4.0% |
| 80 (n=683) |  | | |
| Unadjusted | 1.403(-3.715 to 6.520) | 0.591 | -0.1% |
| Model 1 | 1.606(-3.478 to 6.689) | 0.535 | 0.5% |
| 85 (n=355) |  | | |
| Unadjusted | -4.161(-8.982 to 0.661) | 0.091 | 0.5% |
| Model 1 | -4.891(-9.936 to 0.153) | 0.057 | 2.7% |

*Model 1 is adjusted for smoking, hsCRP and steroid treatment.*

*Model 2 is adjusted for TSH, hsCRP, steroid treatment and smoking at age 75*.

*𝛃 ̂ = regression coefficient*

*CI = confidence interval*

*R^2^=adjusted partial explained variation
*=Muscle mass is defined as ALM-index, appendicular lean mass(kg/height^2^).*
