# Supplementary material for: Body composition has limited influence on the eGFRcys/eGFRcrea ratio in older women
Source: Clin Kidney J. 2026 Oct 28;19(11):sfag379. doi: 10.1093/ckj/sfag379 (PMC13748965; doi:10.1093/ckj/sfag379)
Supplement: Supplementary file 1 [file sfag379_supplemental_files.zip › Supplementary Table 3 (CKD-EPI).docx]

***Supplementary Table 3a.*** Sensitivity analysis using the CKD-EPI equation to calculate the eGFR ratio. Linear regression of eGFRcys/eGFR_crea_ ratio and body composition (fat mass and muscle mass), muscle strength and sarcopenia. 75.

| *Fat mass (kg)* | | | |
| --- | --- | --- | --- |
| *Age* | *𝛃 ̂ (95% CI)* | *p-value* | *R^2^* |
| 75 (n=963) |  | | |
| Unadjusted | -0.0027(-0.0043 to -0.0010) | 0.001 | 1.1% |
| Model 1 | -0.0025(-0.0041 to -0.00082) | 0.003 | 3.8% |
| Model 2 | -0.0029(-0.0046 to -0.0011) | 0.001 | 5.3% |
| 80 (n=683) |  | | |
| Unadjusted | -0.0021(-0.0035 to -0.00071) | 0.003 | 1.2% |
| Model 1 | -0.0022(-0.0036 to -0.00079) | 0.002 | 5.4% |
| 85 (n=355) |  | |  |
| Unadjusted | -0.0010(-0.0030 to 0.0010) | 0.318 | 0.0002% |
| Model 1 | -0.00093(-0.0030 to 0.0012) | 0.399 | 1.3% |
| *Muscle mass (kg/height^2^)** | | | |
| *Age* | *𝛃 ̂ (95% CI)* | *p-value* | *R^2^* |
| 75 (n=963) |  | | |
| Unadjusted | 0.032 (0.012 to 0.052) | 0.002 | 1.0% |
| Model 1 | 0.030 (0.010 to 0.049) | 0.003 | 3.8% |
| Model 2 | 0.029(0.009 to 0.050) | 0.005 | 5.0% |
| 80 (n=683) |  | | |
| Unadjusted | 0.021 (0.006 to 0.042) | 0.027 | 0.6% |
| Model 1 | 0.024 (0.002 to 0.039) | 0.011 | 5.0% |
| 85 (n=355) |  | |  |
| Unadjusted | 0.019 (-0.009 to 0.046) | 0.190 | 0.2% |
| Model 1 | 0.016 (-0.014 to 0.045) | 0.289 | 1.5% |
| *Muscle strength (Nm)* | | | |
| *Age* | *𝛃 ̂ (95% CI)* | *p-value* | *R^2^* |
| 75 (n=963) |  | | |
| Unadjusted | 0.001(0.4 x10^-3^ to 0.001) | <0.001 | 5.8% |
| Model 1 | 0.001(0.4 x10^-3^ to 0.001) | <0.001 | 8.3% |
| Model 2 | 0.001(0.4 x10^-3^ to 0.001) | <0.001 | 9.7% |
| 80 (n=683) |  |  |  |
| Unadjusted | 0.4 x10^-3^(0.3 x10^-3^ to 0.001) | <0.001 | 5.2% |
| Model 1 | 0.4 x10^-3^(0.3 x10^-3^ to 0.001) | <0.001 | 7.1% |
| 85 (n=355) |  |  |  |
| Unadjusted | 0.4 x10^-3^(0.2 x10^-3^ to 0.001) | 0.001 | 3.3% |
| Model 1 | 0.4 x10^-3^(0.1 x10^-3^ to 0.001) | 0.006 | 2.5% |
| *Sarcopenia* | | | |
| *Age* | *𝛃 ̂ (95% CI)* | *p-value* | *R^2^* |
| 75 (n=963) |  | | |
| Unadjusted | -0.10 (-0.18 to -0.03) | 0.007 | 0.7% |
| Model 1 | -0.10 (-0.17 to -0.03) | 0.007 | 4.1% |
| Model 2 | -0.10(-0.17 to -0.03) | 0.008 | 5.3% |
| 80 (n=683) |  | | |
| Unadjusted | -0.036 (-0.088 to 0.016) | 0.178 | 0.1% |
| Model 1 | -0.035 (-0.087 to 0.016) | 0.175 | 3.1% |
| 85 (n=355) |  | | |
| Unadjusted | - 0.07 (-0.13 to -0.02) | 0.0012 | 1.6% |
| Model 1 | -0.08 (-0.1 to -0.02) | 0.014 | 1.8% |

*Model 1 is adjusted for smoking, hsCRP and steroid treatment.*

*Model 2 is adjusted for TSH, hsCRP, steroid treatment and smoking at age 75*.

*𝛃 ̂ = regression coefficient*

*CI = confidence interval*

*R^2^=adjusted partial explained variation
*=Muscle mass is defined as ALM-index, appendicular lean mass(kg/height^2^).*

***Supplementary Table 3b.*** Sensitivity analysis using the CKD-EPI equation to calculate the eGFR_crea_. Linear regression of eGFR_crea_ and body composition (fat mass and muscle mass), muscle strength and sarcopenia.

| *Fat mass (kg)* | | | |
| --- | --- | --- | --- |
| *Age* | *𝛃 ̂ (95% CI)* | *p-value* | *R^2^* |
| 75 (n=963) |  | | |
| Unadjusted | -0.21(-0.31 to -0.10) | <0.001 | 1.6% |
| Model 1 | -0.21(-0.32 to -0.11) | <0.001 | 1.9% |
| Model 2 | -0.19(-0.30 to -0.080) | <0.001 | 1.9% |
| 80 (n=683) |  | | |
| Unadjusted | -0.36(-0.49 to -0.23) | <0.001 | 4.2% |
| Model 1 | -0.36(-0.49 to -0.22) | <0.001 | 3.7% |
| 85 (n=355) |  | |  |
| Unadjusted | -0.36(-1.00 to -0.17) | <0.001 | 3.6% |
| Model 1 | -0.38(-1.00 to -0.19) | <0.001 | 5.3% |
| *Muscle mass (kg/height^2^)** | | | |
| *Age* | *𝛃 ̂ (95% CI)* | *p-value* | *R^2^* |
| 75 (n=963) |  | | |
| Unadjusted | -0.985(-2.26 to 0.289) | 0.129 | 0.1% |
| Model 1 | -1.149(-2.441 to 0.144) | 0.081 | 0.5% |
| Model 2 | -0.902(-2.258 to 0.453) | 0.192 | 0.8% |
| 80 (n=683) |  | | |
| Unadjusted | -1.874(-3.640 to -0.108) | 0.038 | 0.5% |
| Model 1 | -1.640 (-3.442 to 0.162) | 0.074 | -0.01% |
| 85 (n=355) |  | |  |
| Unadjusted | -1.855(-4.459 to 0.749) | 0.162 | 0.3% |
| Model 1 | -1.928(-4.586 to 0.731) | 0.155 | 1.7% |
| *Muscle strength (Nm)* | | | |
| *Age* | *𝛃 ̂ (95% CI)* | *p-value* | *R^2^* |
| 75 (n=963) |  | | |
| Unadjusted | -0.001(-0.011 to 0.010) | 0.923 | -0.1% |
| Model 1 | -0.003(-0.013 to 0.008) | 0.636 | 0.4% |
| Model 2 | -0.002(-0.013 to 0.009) | 0.686 | 0.9% |
| 80 (n=683) |  |  |  |
| Unadjusted | -0.010(-0.026 to 0.007) | 0.252 | 0.1% |
| Model 1 | -0.007(-0.023 to 0.010) | 0.420 | -0.5% |
| 85 (n=355) |  |  |  |
| Unadjusted | -0.00023(-0.027 to 0.027) | 0.987 | -0.4% |
| Model 1 | -0.004(-0.033 to 0.026) | 0.807 | 1.4% |
| *Sarcopenia* | | | |
| *Age* | *𝛃 ̂ (95% CI)* | *p-value* | *R^2^* |
| 75 (n=963) |  | | |
| Unadjusted | -1.26(-6.01 to 3.49) | 0.603 | -0.1% |
| Model 1 | -1.18(-5.94 to 3.58) | 0.626 | 0.1% |
| Model 2 | -1.22(-5.99 to 3.56) | 0.617 | 0.5% |
| 80 (n=683) |  | | |
| Unadjusted | 3.985(-0.989 to 8.959) | 0.116 | 0.2% |
| Model 1 | 4.744(-0.295 to 9.784) | 0.065 | -0.01% |
| 85 (n=355) |  | | |
| Unadjusted | 0.639(-4.671 to 5.949) | 0.813 | -0.3% |
| Model 1 | 0.033(-5.546 to 5.611) | 0.991 | 1.6% |

*Model 1 is adjusted for smoking, hsCRP and steroid treatment.*

*Model 2 is adjusted for TSH, hsCRP, steroid treatment and smoking at age 75*.

*𝛃 ̂ = regression coefficient*

*CI = confidence interval*

*R^2^=adjusted partial explained variation
*=Muscle mass is defined as ALM-index, appendicular lean mass(kg/height^2^).*

***Supplementary Table 3c.*** Sensitivity analysis using the CKD-EPI equation to calculate the eGFR_cys_. Linear regression of eGFR_cys_ and body composition (fat mass and muscle mass), muscle strength and sarcopenia.

| *Fat mass (kg)* | | | |
| --- | --- | --- | --- |
| *Age* | *𝛃 ̂ (95% CI)* | *p-value* | *R^2^* |
| 75 (n=963) |  | | |
| Unadjusted | -0.38(-1.0 to -0.24) | <0.001 | 2.9% |
| Model 1 | -0.38(-1.0 to -0.23) | <0.001 | 6.0% |
| Model 2 | -0.39(-1.0 to -0.25) | <0.001 | 6.2% |
| 80 (n=683) |  | | |
| Unadjusted | -0.45(-1.0 to -0.32) | <0.001 | 6.2% |
| Model 1 | -0.46(-1.0 to -0.32) | <0.001 | 7.4% |
| 85 (n=355) |  | |  |
| Unadjusted | -0.35(-1.0 to -0.19) | <0.001 | 4.4% |
| Model 1 | -0.39(-1.0 to -0.22) | <0.001 | 6.8% |
| *Muscle mass (kg/height^2^)** | | | |
| *Age* | *𝛃 ̂ (95% CI)* | *p-value* | *R^2^* |
| 75 (n=963) |  | | |
| Unadjusted | 0.996(-0.754 to 2.746) | 0.264 | 0.02% |
| Model 1 | 0.698(-1.077 to 2.472) | 0.440 | 3.2% |
| Model 2 | 0.903(-0.961 to 2.768) | 0.342 | 3.2% |
| 80 (n=683) |  | | |
| Unadjusted | -0.292(-2.114 to 1.531) | 0.753 | -0.1% |
| Model 1 | 0.111(-1.730 to 1.953) | 0.905 | 1.1% |
| 85 (n=355) |  | |  |
| Unadjusted | -0.368(-2.652 to 1.916) | 0.751 | -0.3% |
| Model 1 | -0.671(-3.069 to 1.726) | 0.582 | 1.3% |
| *Muscle strength (Nm)* | | | |
| *Age* | *𝛃 ̂ (95% CI)* | *p-value* | *R^2^* |
| 75 (n=963) |  | | |
| Unadjusted | 0.040(0.026 to 0.054) | <0.001 | 3.2% |
| Model 1 | 0.036(0.022 to 0.051) | <0.001 | 6.2% |
| Model 2 | 0.037(0.022 to 0.051) | <0.001 | 6.2% |
| 80 (n=683) |  |  |  |
| Unadjusted | 0.024(0.007 to 0.041) | 0.006 | 1.2% |
| Model 1 | 0.025(0.007 to 0.042) | 0.005 | 1.6% |
| 85 (n=355) |  |  |  |
| Unadjusted | 0.024(-0.0004 to 0.048) | 0.054 | 0.9% |
| Model 1 | 0.020(-0.006 to 0.047) | 0.134 | 1.9% |
| *Sarcopenia* | | | |
| *Age* | *𝛃 ̂ (95% CI)* | *p-value* | *R^2^* |
| 75 (n=963) |  | | |
| Unadjusted | -8.672(-15.111 to -2.234) | 0.008 | 0.7% |
| Model 1 | -8.424(-14.892 to -1.957) | 0.011 | 4.0% |
| Model 2 | -8.382(-14.879 to -1.885) | 0.012 | 4.0% |
| 80 (n=683) |  | | |
| Unadjusted | 1.438(-3.758 to 6.635) | 0.587 | -0.1% |
| Model 1 | 1.653(-3.505 to 6.812) | 0.529 | 0.6% |
| 85 (n=355) |  | | |
| Unadjusted | -4.015(-8.734 to 0.704) | 0.095 | 0.5% |
| Model 1 | -4.736(-9.682 to 0.210) | 0.060 | 2.5% |

*Model 1 is adjusted for smoking, hsCRP and steroid treatment.*

*Model 2 is adjusted for TSH, hsCRP, steroid treatment and smoking at age 75*.

*𝛃 ̂ = regression coefficient*

*CI = confidence interval*

*R^2^=adjusted partial explained variation
*=Muscle mass is defined as ALM-index, appendicular lean mass(kg/height^2^).*
